# Different Symbiotic Species of *Armillaria* Affect the Yield and Active Compound Contents of *Polyporus umbellatus*

**DOI:** 10.3390/microorganisms13020228

**Published:** 2025-01-22

**Authors:** Liu Liu, Yongmei Xing, Shoujian Li, Lisi Zhou, Bing Li, Shunxing Guo

**Affiliations:** 1 Institute of Medicinal Plant Development, Chinese Academy of Medical Sciences and Peking Union Medical College, Beijing 100193, China; liuliu0026@foxmail.com (L.L.); meimary@163.com (Y.X.); 13756298450@163.com (S.L.); zls921203@sina.com (L.Z.); 2 State Key Laboratory of Bioactive Substance and Function of Natural Medicines, Beijing 100193, China

**Keywords:** *Polyporus umbellatus*, *Armillaria*, ergosterol, polyporusterone A, polyporusterone B, polysaccharides

## Abstract

*Polyporus umbellatus* is a medicinal fungus primarily used for diuresis, with its sclerotium serving as the medicinal component. The growth and development of sclerotia are reliant on a symbiotic relationship with *Armillaria*. However, the impact of different *Armillaria* species on the yield and quality of sclerotia remains unclear. In this study, three *Armillaria* strains, A35, A541, and A19, were identified through TEF-1α sequence analysis and phylogenetic classification. These strains were classified into three distinct species: A35 as *A. ostoyae*, A541 as *A. gallica*, while the taxonomic status of A19 remains unresolved. After four years of co-cultivation with these *Armillaria* strains, three groups of *P. umbellatus* sclerotia were harvested and labeled as A35-P, A541-P, and A19-P, respectively. The yields of A35-P, A541-P, and A19-P exhibited significant variations, with A541-P achieving the highest yield (1221 ± 258 g·nest^−1^), followed by A35-P (979 ± 201 g·nest^−1^), and A19-P yielding the least (591 ± 54 g·nest^−1^). HPLC revealed significant differences in the levels of polyporusterone A and polyporusterone B among the groups. The total polysaccharide content, determined via the phenol-sulfuric acid method, also varied significantly, with A541-P recording the highest content (0.897 ± 0.042%), followed by A19-P (0.686 ± 0.058%), and A35-P showing the lowest value (0.511 ± 0.083%). PCA based on these data indicated clear distinctions among A35-P, A541-P, and A19-P, with the three groups forming separate clusters. This study, for the first time, demonstrates the effects of three different *Armillaria* species on the yield and active compound content of *P. umbellatus*. These findings provide valuable insights for selecting high-quality *Armillaria* strains and offer guidance for the artificial cultivation of *P. umbellatus*.

## 1. Introduction

*Polyporus umbellatus* (Pers.) Fries is a fungus belonging to the Polyporaceae family, consisting of two primary parts: the sclerotium and the fruiting body (commonly referred to as mushrooms). The fruiting body, which emerges from the underground sclerotium, is the edible portion. The sclerotium, found beneath the soil, has been recognized as a valuable component of traditional Chinese medicine for over 2000 years, primarily used for its dampness-clearing and diuretic properties [1]. Modern pharmacological research has validated the diuretic [2], renal protective [3], anti-tumor [4], and other beneficial activities of *P. umbellatus* [5]. The bioactive compounds identified in *P. umbellatus* are predominantly steroids and polysaccharides [6]. Over 30 steroidal compounds have been isolated and characterized from their sclerotium, fruiting body, and fermenting mycelium [7,8]. For instance, ergosterol and ergosta-4,6,8(14),22-tetraen-3-one, extracted from the sclerotia, have demonstrated diuretic effects [9]. Additional steroids, such as polyporusterone A and polyporusterone B, have exhibited cytotoxicity against leukemia 1210 cells [10], antioxidant activity by inhibiting 2,2-azino-bis(2-aminopropane)dichloride-induced erythrocyte lysis [11], and hair growth-promoting properties [12]. In the *Chinese Pharmacopoeia* (ChP, 2020), ergosterol is designated as an index compound for evaluating the quality of *P. umbellatus* sclerotia, with a stipulated minimum content of 0.070%. Polysaccharides, another significant class of active compounds in *P. umbellatus*, have demonstrated diverse pharmacological activities, including immunomodulatory, anti-tumor, hepatoprotective, and antiradiation effects in in vivo and in vitro studies [13,14].

Given the efficacy of *P. umbellatus* in treating edema and supporting anti-tumor activities, clinical applications of drugs derived from *P. umbellatus*, such as the *P. umbellatus* polysaccharide injection and Wuling-san, have become widespread [15]. Consequently, the artificial cultivation of *P. umbellatus* is essential to meet market demand and conserve wild *P. umbellatus* resources. However, the success of artificial cultivation is influenced by factors such as the *Armillaria* species and environmental conditions, leading to challenges with inconsistent yields and variable quality. The growth and development of *P. umbellatus* sclerotia are dependent on a special symbiotic relationship with *Armillaria*, a genus within the family Physalacriaceae [16]. Under natural conditions, there is a special symbiotic relationship between *P. umbellatus* and *Armillaria*. *Armillaria* invades *P. umbellatus* via its rhizomorphs, triggering the innate immune response of *P. umbellatus* to resist foreign infection. During this process, the mycelial cells of *P. umbellatus* undergo lignification, forming isolation cavities resembling the structure of the sclerotia epidermis. These cavities encapsulate the hyphae of both *Armillaria* and *P. umbellatus*. Within the isolation cavities, *Armillaria* digests the separated mycelium of *P. umbellatus*. Simultaneously, the *P. umbellatus* mycelium can invade or attach to the *Armillaria* mycelium and its infection zone’s intercellular spaces to absorb metabolites. This interaction enables the growth of *P. umbellatus* mycelium and its differentiation into new sclerotia [17]. In artificial cultivation, uncertainties surrounding the source and species of *Armillaria* can lead to non-growth or the “empty nest” phenomenon, presenting a major limitation for the development of the *P. umbellatus* industry. Previous studies indicated that infestation with *Armillaria* increased the ergosterol content in *P. umbellatus* from 0.0632 to 0.0809%, an improvement of 28%. Additionally, the polysaccharide content rose from 3.69 to 5.45%, representing a 47.7% increase [18]. Further research involving 28 strains of *Armillaria* isolated from wild *P. umbellatus* identified *A. mellea*, *A. gallica*, *A. cepistipes*, and *A. ostoyae* as potential symbiotic partners, indicating that *P. umbellatus* exhibits low selectivity for symbiotic *Armillaria* species [19]. Despite this, it remains unclear which specific *Armillaria* species are optimal for co-cultivation with *P. umbellatus* to improve yields and quality.

In this study, three *Armillaria* strains, A19, A35, and A541, were selected for co-cultivation with *P. umbellatus*. These strains, identified and grouped into distinct *Armillaria* clades through molecular methods, were used to examine their impact on the yield and contents of ergosterol, polyporusterone A, polyporusterone B, and polysaccharides in *P. umbellatus*. The findings provide a reference for selecting the most suitable *Armillaria* strains for the artificial cultivation of *P. umbellatus*.

## 2. Materials and Methods

### 2.1. Strain and Culture of Armillaria

Three *Armillaria* strains were isolated and purified from wild *P. umbellatus* sclerotia. These strains were preserved at the Institute of Medicinal Plants, Chinese Academy of Medical Sciences, under preservation numbers A35, A541, and A19. A541 and A35 were isolated from wild *P. umbellatus* in Liuba and Lueyang, Shaanxi Province, respectively, while A19 was isolated from *P. umbellatus* in Zhaotong, Yunnan Province. A35, A541, and A19 were cultured on a potato dextrose agar (PDA) medium (1 L of medium consisting of 200 g of potato, 10 g of dextrose, and 12 g of agar) at room temperature in the dark.

For co-cultivation with *P. umbellatus*, the *Armillaria* strains on PDA plates were further cultured to spawn due to their rapid growth, which facilitated infection of *P. umbellatus* and simplified contamination control and transportation. Tree branches from the Fagaceae family were selected as a growth substrate for the *Armillaria* spawn because of their well-developed sapwood and moderately thick bark [20]. Chestnut tree branches approximately 2 cm in diameter and 5 cm in length were soaked in water for 24 h, bottled, and then submerged in water. The bottles were sterilized, creating a medium suitable for inoculation with *Armillaria* strains under aseptic conditions. The *Armillaria* strains were incubated in this medium at room temperature in the dark for 40–50 days until the medium was fully colonized [21]. These cultures were subsequently used for co-cultivation with *P. umbellatus*.

### 2.2. DNA Extraction, PCR Amplification, and Sequencing of A35, A541, and A19

Fresh rhizomorphs of A35, A541, and A19, cultured on PDA for approximately 15 days, were collected and ground into powder using liquid nitrogen to facilitate DNA extraction. DNA was extracted following the instructions provided in the DNA extraction kit (Aidlab, Beijing, China). The partial TEF-1α (transcription elongation factor) gene was amplified using the primer pair TEF-F (5′-GGCATCGAGGAGAGAGTCTTG-3′) and TEF-R (5′-TATCTCCAAGGACGGGCAGA-3′) [22]. The PCR amplification system consisted of 12.5 μL of 2× Taq Master Mix (Vazyme, Nanjing, China), 1 μL of each primer (10 μmol·L^−1^), 2 μL of DNA template, and 8.5 μL of ddH_2_O, for a total reaction volume of 25 μL. The reaction conditions were as follows: initial denaturation at 95 °C for 3 min, followed by 34 cycles of denaturation at 95 °C for 15 s, annealing at 60 °C for 15 s, and extension at 72 °C for 60 s, with a final extension step at 72 °C for 5 min. The PCR products were sequenced by RuiBiotech Co., Ltd., Beijing, China.

### 2.3. Nucleotide Alignment and Phylogenetic Analysis

The sequences obtained from PCR products were analyzed using the BLAST tool at the National Center for Biotechnology Information (NCBI) to identify sequences with high homology. These homologous sequences were downloaded and used to construct a phylogenetic tree with MEGA 7.0 software [23]. *Lentinula edodes* was designated as the outgroup for the analysis. The evolutionary distance was calculated using the maximum likelihood method, and the confidence levels of the branching points were assessed through bootstrap analysis with 1000 replicates.

### 2.4. Artificial Co-Cultivation Experiment of P. umbellatus

The artificial co-cultivation experiment was carried out between October 2018 and March 2022 in Liuba County, Shaanxi Province, a major production area of *P. umbellatus* in China. The experimental site was situated at an altitude of approximately 1450 m, featuring forested, gently sloping terrain with well-drained, humus-rich soil. The co-cultivation methodology followed previously reported protocols [24] with modifications to certain parameters. Nests were dug along the slope, each with dimensions of approximately 30 cm × 30 cm (depth × width). A layer of thin leaves, about 2 cm thick, was spread on the base of each nest. Two segments of tree sticks, each with a diameter of 8–12 cm, were placed side by side with a spacing of 3–5 cm. Each nest was then filled with 250 g of *P. umbellatus* sclerotia (fresh, tender, and highly viable seeds purchased from Qinzheng Zhuling Development Co., Ltd. in Liuba County, Hanzhong, China), 500 g of small branches, and 1 bottle of *Armillaria* spawn. The materials were positioned on both sides and on top of the tree sticks. A thin layer of humus soil and leaves was sprinkled over the materials, followed by a 5 cm layer of soil to cover the nest. At least 30 nests of *P. umbellatus* were co-cultivated with each strain of *Armillaria*. The nests were spaced 15 cm apart, and after four years of cultivation, the *P. umbellatus* was harvested. The samples of *P. umbellatus* co-cultivated with A35, A541, and A19 were designated as A35-P, A541-P, and A19-P, respectively.

### 2.5. Sample Collection and Yield Comparisons of P. umbellatus Sclerotia

The *P. umbellatus* sclerotia were harvested in the fourth year of co-cultivation. Surface sediment, residual humus, and other impurities were carefully removed from the sclerotia before weighing. To determine the yield of *P. umbellatus* after co-cultivation, at least eight nests were dug up from each group. The average yield from each group was calculated, and the differences in yield among the three groups, A35-P, A541-P, and A19-P, were compared.

### 2.6. High-Performance Liquid Chromatography for Detecting Ergosterol, Polyporusterone A, and Polyporusterone B in P. umbellatus Sclerotia

The reference substance for ergosterol was obtained from Beijing Solarbio Science & Technology Co., Ltd., Beijing, China, while the reference substances for polyporusterone A and polyporusterone B were purchased from Sichuan Scvictory Biotechnology Co., Ltd. (Chengdu, China). The reference substances—ergosterol, polyporusterone A, and polyporusterone B—were accurately weighed at 1.47 mg, 1.57 mg, and 1.57 mg, respectively. Each was diluted in methanol within a volumetric flask to a precise volume of 2 mL, resulting in concentrations of 0.735, 0.785, and 0.785 mg·mL^−1^, respectively. Different volumes of the single-reference solutions were combined to prepare a mixed-reference solution for qualitative analysis.

Fresh *P. umbellatus* sclerotia samples from the A19-P, A35-P, and A541-P groups (three samples per group) were collected, washed, and sun-dried. The samples were further dried in an oven at 60 °C until their weight stabilized. The dried samples were then ground into powder and passed through a 40-mesh sieve. From each powdered sample, 3 g was accurately weighed, and 20 volumes of 95% ethanol were added. The samples were soaked in ethanol for 12 h and subsequently subjected to ultrasonication for 1 h. The extracts were filtered and concentrated using rotary evaporation. The resulting concentrate was transferred to an evaporating dish, where the remaining ethanol was allowed to volatilize at room temperature. The residue was redissolved in methanol, and the solution was transferred into a volumetric flask with a final volume of 2 mL [25,26]. Each sample group was processed in triplicate to ensure reproducibility.

High-performance liquid chromatography (HPLC) analysis was conducted using the 1260 Infinity II LC System (Agilent, Santa Clara, CA, USA), which includes a quadruple pump, autosampler, and variable wavelength ultraviolet detector. The experiments utilized a Bridge RP18 column (250 mm × 4.6 mm, 5 μm, Waters, Milford, MA, USA). The flow rate was maintained at 1.0 mL·min^−1^, and the column temperature was set to room temperature. The detection wavelength was configured as follows: 247 nm for the first 25 min, suitable for detecting polyporusterone A and polyporusterone B, and 283 nm after 25 min for ergosterol detection. Both standard and sample solutions were filtered through a 0.22 μm organic phase filter membrane prior to injection, and 20 μL of each sample solution was injected into the HPLC system. The mobile phase consisted of the following components: A (acetonitrile), B (water), and D (methanol). The elution program was as follows: 0–25 min, A%:B% = 78:22; 25–42 min, 100% D; and 42–45 min, A%:B% = 78:22 [25,26].

### 2.7. Phenol-Sulfuric Acid Method for Detecting the Polysaccharides in P. umbellatus

The phenol-sulfuric acid method was employed to quantify the polysaccharides in *P. umbellatus* sclerotia. For the preparation of the reference standard, 10 mg of glucose was accurately weighed and diluted with methanol in a volumetric flask to a final volume of 10 mL. From this solution, volumes of 0, 0.1, 0.2, 0.3, 0.4, 0.5, 0.6, 0.7, 0.8, 0.9, and 1.0 mL were precisely measured into separate test tubes, and the volume in each tube was adjusted to 2.0 mL with water. Next, 1 mL of freshly prepared 5% phenol solution and 5 mL of concentrated sulfuric acid were added to each test tube. The mixtures were shaken thoroughly, allowed to stand at room temperature for 10 min, and then maintained at 40 °C for 15 min. Afterward, they were cooled in an ice bath, and the absorbance was measured at 490 nm. A standard curve was generated by plotting absorbance (Y-axis) against the glucose reference concentration (X-axis).

To prepare the test solution, powdered *P. umbellatus* sclerotia from the A19-P, A35-P, and A541-P samples were used. For each sample, 3 g of sclerotia powder was accurately weighed, and 20 mL of ultrapure water was added. The mixture was subjected to ultrasonication for 1 h, and the extract was concentrated under reduced pressure, with the weight recorded. Subsequently, three times the volume of 85% ethanol was added to the concentrate, and the mixture was stored at 4 °C overnight. The mixture was centrifuged for 30 min, and the supernatant was discarded. The precipitate was dissolved in water to produce the test solution, which was appropriately diluted and analyzed using the same procedure described for the standard curve. The total polysaccharide content in *P. umbellatus* was calculated based on the standard curve [27]. Each sample group was analyzed in triplicate to ensure accuracy.

### 2.8. Statistical Analysis

The experimental data on the percentage content of the active compounds in *P. umbellatus* were calculated on a dry weight basis and expressed as mean ± standard deviation. Statistical analyses were conducted using IBM SPSS 25.0 software (IBM Corp., Armonk, NY, USA), with ANOVA, followed by LSD tests to determine the statistical significance, where *p* < 0.05 was considered significant. Data related to the yield, ergosterol, polyporusterone A, polyporusterone B, and polysaccharide contents of A35-P, A541-P, and A19-P were processed and analyzed using GraphPad Prism 9.5 (GraphPad Software, San Diego, CA, USA) via principal component analysis (PCA). PCA was performed on standardized data, and PCs with eigenvalues greater than 1 were selected for further interpretation.

## 3. Results

### 3.1. A35, A541, and A19 Belong to Three Different Species of Armillaria

**Figure 1:**
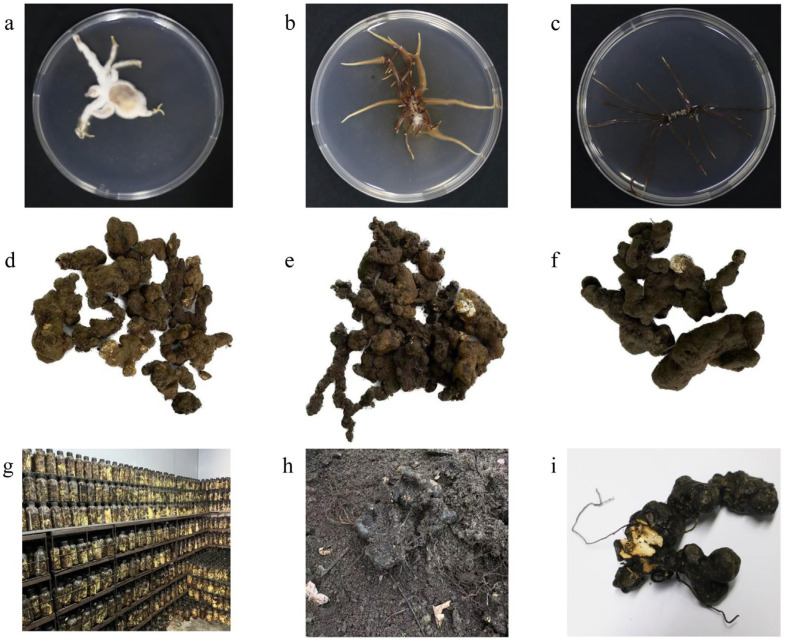
Colony morphology of strains A35, A541, and A19 on PDA medium after 15 days, and *P. umbellatus* sclerotia (A35-P, A541-P, and A19-P) obtained after four years of co-cultivation: (**a**) A35 grown on PDA medium at room temperature in the dark for 2 weeks; (**b**) A541 grown on PDA medium under the same conditions; (**c**) A19 grown on PDA medium under identical conditions; (**d**) A35-P sclerotia excavated from a nest of *P. umbellatus* co-cultivated with A35 for four years; (**e**) A541-P sclerotia excavated from a nest co-cultivated with A541 for four years; (**f**) A19-P sclerotia excavated from a nest co-cultivated with A19 for four years; (**g**) *Armillaria* spawn; (**h**) *P. umbellatus* co-cultivated with *Armillaria* in soil; and (**i**) *Armillaria* infecting *P. umbellatus*.

**Figure 2:**
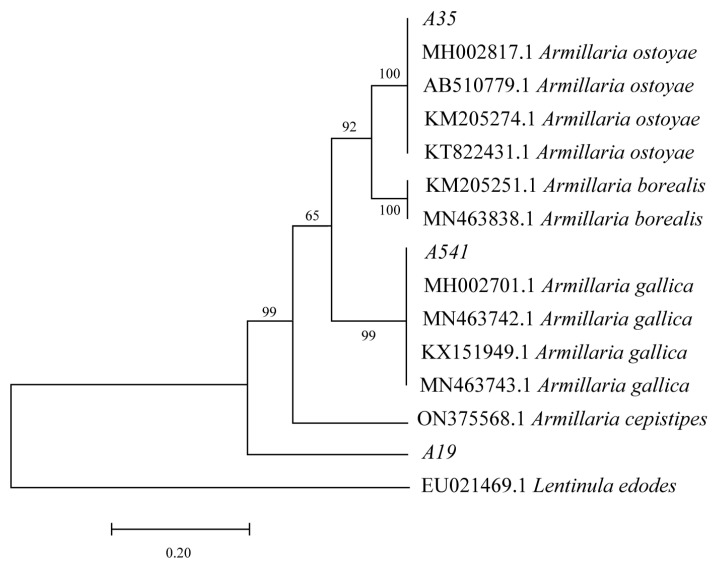
Molecular phylogenetic analysis was performed using the maximum likelihood method based on TEF-1α sequences. Bootstrap support values (from 1000 replicates) are displayed above the branches, representing the confidence level for each branching node.

After 15 days of cultivation on the PDA medium, A35, A541, and A19 exhibited distinct differences in growth patterns and rhizomorph characteristics (Figure 1a–c). A541 displayed the longest and most extensively branched fungal rhizomorphs, while the rhizomorphs of A35 were predominantly surrounded by hyphae. The DNA sequences of A35, A541, and A19 were analyzed using BLAST based on the NCBI database. Sequences with over 95% similarity to the queried sequences were downloaded for phylogenetic analysis. Non-comparable regions at both sequence ends were trimmed using MEGA 7.0 software, and the final aligned sequences were employed to construct a phylogenetic tree (Figure 2). The phylogenetic analysis of the TEF-1α sequences revealed that A35, A541, and A19 clustered into separate branches. A35 and A541 were grouped into the A. ostoyae and A. gallica clades, respectively, while A19 could not be clustered with other species and was phylogenetically distinct from the rest.

### 3.2. Significant Differences in Yield of P. umbellatus Sclerotia After Co-Cultivation with A35, A541, and A19

**Figure 3:**
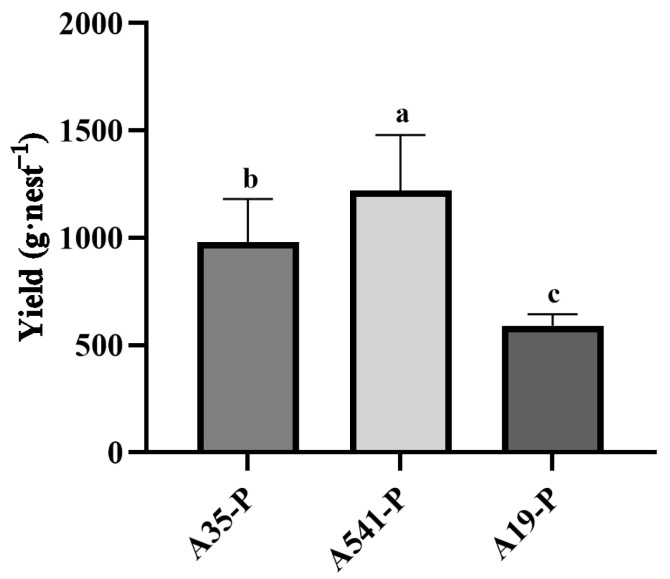
Statistical analysis differences in yield (g·nest^−1^) of A35-P, A541-P, and A19-P. Different lowercase letters show significant differences at the *p* < 0.05 level.

A35, A541, and A19 were all capable of forming a symbiotic relationship with the sclerotia of *P. umbellatus*. By the fourth year of cultivation, new sclerotia had successfully germinated and developed into mature medicinal materials (Figure 1d–f). Upon co-cultivation with A35, the yield of fresh sclerotia reached (979 ± 201) g·nest^−1^ (n = 12), starting from an initial seedling weight of 250 g. Co-cultivation with A541 resulted in a yield of (1221 ± 258) g·nest^−1^ (n = 15), while co-cultivation with A19 produced (591 ± 54) g·nest^−1^ (n = 8). Variance analysis (ANOVA) revealed significant differences in the yields of *P. umbellatus* sclerotia among the groups cultivated with A35, A541, and A19 (Figure 3).

### 3.3. Validation of Standard Curves and HPLC Quantification Methods

**Figure 4:**
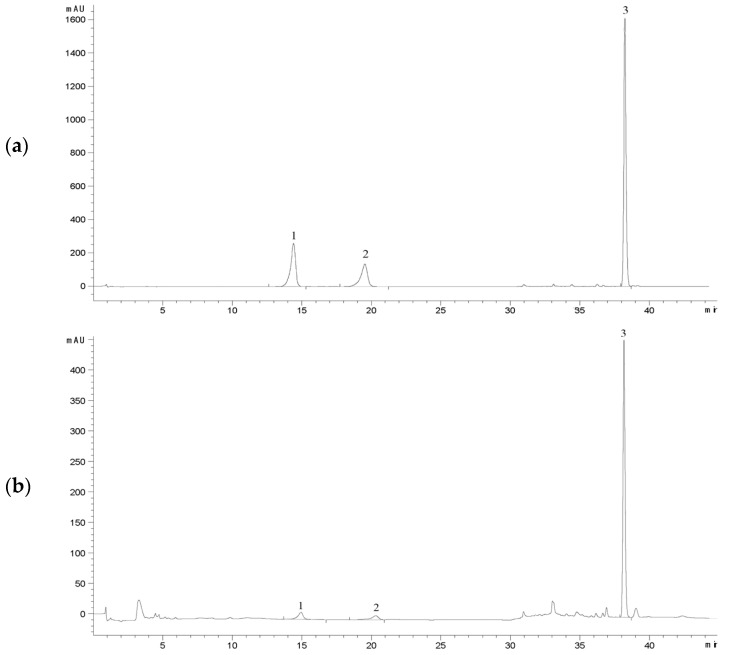
HPLC chromatograms for the determination of ergosterol, polyporusterone A, and polyporusterone B contents in *P. umbellatus* samples. (**a**) Chromatogram of the reference substances: polyporusterone B (1), polyporusterone A (2), and ergosterol (3); (**b**) chromatogram of substances 1–3 detected in *P. umbellatus* samples. The detection wavelength was set to 247 nm for the first 25 min and switched to 283 nm after 25 min.

A HPLC method was established for the quantification of ergosterol, polyporusterone A, and polyporusterone B. Validation of the method confirmed that its precision and accuracy met the requirements of the study. The chromatographic peaks for the three target compounds were sharp and symmetrical, with clear separation from one another and no interference from impurities. These attributes were consistent across both the reference standards and all tested samples. The retention times of the components in the test solution closely matched those of the mixed-reference standards (Figure 4).

The RSD values for the peak areas of ergosterol, polyporusterone A, and polyporusterone B were 0.62%, 1.10%, and 0.65%, respectively, for six consecutive injections of the sample solution, indicating that the instrument exhibited good precision.

Linear regression analysis was conducted by plotting the concentration of the reference solution on the horizontal axis (X-axis) and the peak area on the vertical axis (Y-axis). The results demonstrated strong linear relationships for all three reference substances within the experimental concentration ranges. The limits of detection (LOD) and limits of quantification (LOQ) for the three components are presented in Table 1.

The RSD values for the peak areas of ergosterol, polyporusterone A, and polyporusterone B were 1.49%, 1.95%, and 0.55%, respectively, indicating that the test solution exhibited good stability within 24 h. Additionally, the RSD values for these compounds, prepared using the same method, were 0.80%, 1.10%, and 1.60%, respectively, demonstrating good reproducibility. The average recoveries of ergosterol, polyporusterone A, and polyporusterone B were 102.74%, 97.90%, and 101.12%, with corresponding RSD values of 1.53%, 4.78%, and 1.59%, confirming the method’s accuracy.

### 3.4. The Ergosterol, Polyporusterone A, and Polyporusterone B Percentage of Contents of P. umbellatus

**Figure 5:**
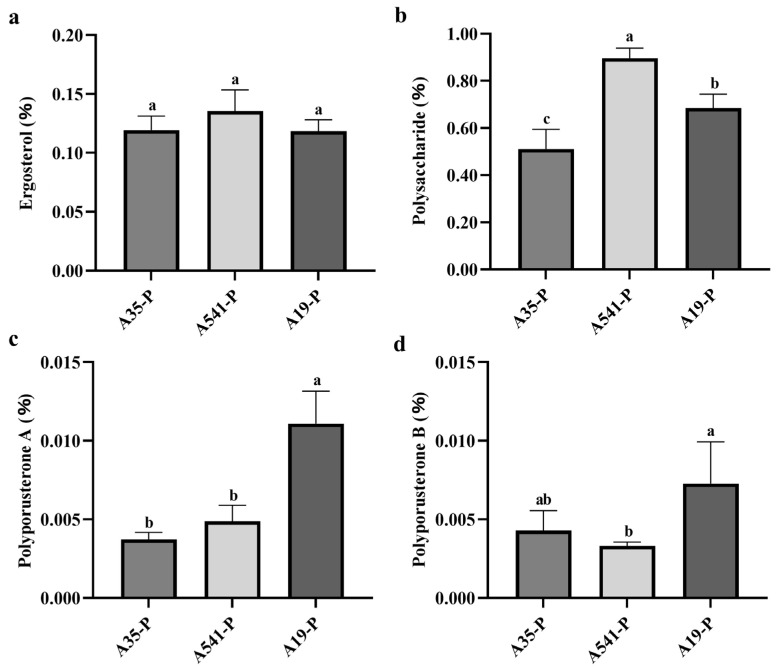
Statistical analysis of ergosterol, polysaccharide, polyporusterone A, and polyporusterone B contents in A35-P, A541-P, and A19-P. (**a**) Ergosterol content (%) in A35-P, A541-P, and A19-P; (**b**) polysaccharide content (%) in A35-P, A541-P, and A19-P; (**c**) polyporusterone A content (%) in A35-P, A541-P, and A19-P; and (**d**) polyporusterone B content (%) in A35-P, A541-P, and A19-P. Different lowercase letters indicate significant differences at the *p* < 0.05 level.

The ergosterol, polyporusterone A, and polyporusterone B contents in the samples were calculated based on the standard curve. The ergosterol content in A35-P, A541-P, and A19-P was 0.119 ± 0.012%, 0.136 ± 0.018%, and 0.118 ± 0.010%, respectively (Figure 5a). These values exceeded the threshold specified in the ChP, indicating that the cultivated sclerotia met the medicinal quality standards. Variance analysis showed differences in the ergosterol content among A35-P, A541-P, and A19-P, but these differences were not statistically significant (Figure 5a). The polyporusterone A content was 0.004 ± 0.0004%, 0.005 ± 0.001%, and 0.011 ± 0.002% in A35-P, A541-P, and A19-P, respectively (Figure 5c). The polyporusterone B content was measured at 0.004 ± 0.001%, 0.003 ± 0.0003%, and 0.007 ± 0.003% in A35-P, A541-P, and A19-P, respectively (Figure 5d). There was no significant difference in polyporusterone A content between A35-P and A541-P, but both were significantly lower than the content in A19-P. Similarly, the polyporusterone B content was highest in A19-P, with a statistically significant difference compared to A541-P, which exhibited the lowest value. The polyporusterone B content in A35-P did not significantly differ from either A19-P or A541-P.

### 3.5. Total Polysaccharide Contents of P. umbellatus

The total polysaccharide content of *P. umbellatus* was calculated based on the standard curve y = 4.2455x + 0.0323y = 4.2455x + 0.0323y = 4.2455x + 0.0323 (R^2^ = 0.9997). The polysaccharide contents were determined to be 0.511 ± 0.083% for A35-P, 0.897 ± 0.042% for A541-P, and 0.686 ± 0.058% for A19-P (Figure 5b). Variance analysis indicated significant differences in the polysaccharide content among the three groups.

### 3.6. PCA Analysis of P. umbellatus

**Figure 6:**
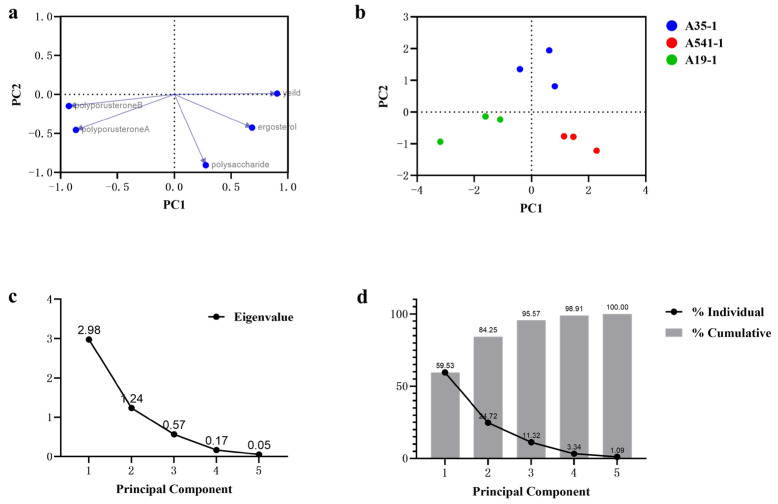
PCA of A35-P, A541-P, and A19-P samples based on yield, ergosterol, polyporusterone A, polyporusterone B, and polysaccharide contents. (**a**) The loading plot depicts the grouping of variables using the first two principal components. Yield, ergosterol, polyporusterone A, and polyporusterone B contents showed strong correlations (values close to 1 or −1) with PC1, while polysaccharide content was primarily correlated with PC2. (**b**) The PC score plot displays the original eigenvalues of each principal component, illustrating clustering patterns for the three sample groups. (**c**) The scree plot indicates that the eigenvalues of PC1 and PC2 were greater than 1, with the variance trend stabilizing after PC3. (**d**) The proportion of variance plot reveals that the first (PC1) accounted for 59.53% of the total variation, while the cumulative variance of PC1 and PC2 explained 84.25% of the total variation.

Principal component analysis (PCA) is widely used for distinguishing and discriminating among sample groups or species [28]. PCA was employed to analyze the relationships and differences in yield, ergosterol, polyporusterone A, polyporusterone B, and polysaccharide contents among A35, A541, and A19. The PCA results separated the yield and active compound contents into two primary components, PC1 and PC2. PC1 explained 59.53% of the total variation, while the cumulative variance of PC1 and PC2 accounted for 84.25% of the total variance (Figure 6d). The eigenvectors (principal component vectors) represent specific linear combinations of the variables. From the scree plot (Figure 6c), the eigenvalues for PC1 and PC2 were greater than 1, and the trend stabilized after PC3. Therefore, PC1 and PC2 were selected for the PCA analysis. The loading plots showed that the yield, ergosterol, polyporusterone A, and polyporusterone B contents were strongly correlated with PC1 (values near 1 or −1), while these variables were less correlated with PC2. In contrast, the polysaccharide content showed a stronger correlation with PC2 (Figure 6a). The PC score plots illustrated the clustering of data points into three distinct groups corresponding to A35, A541, and A19, respectively, based on the first two principal components (Figure 6b).

## 4. Discussion

In recent years, there has been a growing number of reports on *Armillaria* worldwide, with identification methods advancing from traditional morphological approaches to molecular biological techniques [29,30]. The *tef-1α* gene encodes a partial protein called translation elongation factor-1α and is noted for its high sequence polymorphism in closely related species, making it a preferred marker for distinguishing fungal species [31]. Our previous research demonstrated that the TEF-1α sequences of *Armillaria* strains from different sources showed consistent performance, indicating the stability of these sequences across various sources. In contrast, sequence amplification for ITS, IGS, β-tubulin, and LSU exhibited variability among strains from different origins [32]. This stability suggests that TEF-1α sequences are more conserved during the evolution of *Armillaria*, which may explain their current utility in providing high discrimination among species in molecular systematics research. In this study, TEF-1α sequences were employed to identify and analyze three species of *Armillaria* isolated from wild *P. umbellatus*. The results revealed that strain A35 clustered with *A. ostoyae*, while A541 clustered with *A. gallica*. However, A19 formed a separate cluster distinct from several *Armillaria* species, including *A. ostoyae* and *A. gallica*, suggesting that A19, A35, and A541 are distinct species. This finding aligns with Liu et al.’s report that *P. umbellatus* exhibits no selective specialization for symbiotic *Armillaria* species [19,33]. According to Liu et al., four species—*A. cepistipes*, *A. gallica*, *A. mellea*, and *A. ostoyae*—have been reported to form symbiotic relationships with *P. umbellatus*. Consistent with this, the A35 and A541 strains identified in this study clustered with *A. ostoyae* and *A. gallica*, respectively. However, the TEF-1α sequence analysis positioned A19 in a distinct taxonomic cluster. This discrepancy may be attributed to sequencing accuracy or limitations in current identification techniques. Further investigation combining advanced methods will be needed to clarify the taxonomic status of A19.

Similar to the symbiotic relationship between *Armillaria* and *P. umbellatus*, the growth of the orchid plant *Gastrodia elata* (“tianma” in Chinese) also depends on *Armillaria*. Interestingly, the *Armillaria* species used in the cultivation of *G. elata* and *P. umbellatus* sometimes belong to the same species [34]. Previous studies have reported that the yield of *G. elata* cultivated with *Armillaria* strain M1 was 2.04 times higher than that achieved with strain M2 [35]. Similarly, another study demonstrated that the gastrodin content in *G. elata* varied by as much as 1.46 times depending on the *Armillaria* species used [36]. These findings suggest that the distinct characteristics of different *Armillaria* species may result in variations in yield and the contents of active compounds in *P. umbellatus* after co-cultivation. This hypothesis is consistent with our study’s results, which revealed differences in the yield and levels of ergosterol, polyporusterone A, polyporusterone B, and polysaccharides among samples co-cultivated with A35, A541, and A19. All three strains increased the yield of *P. umbellatus*, with A541-P showing the most pronounced effect. This variation could be attributed to the biological characteristics of the three *Armillaria* strains, including the rhizomorph thickness and branching, growth rate, and parasitism capacity [37]. Morphologically, under identical medium and culture conditions, A541 exhibited faster growth and more extensive branching compared to the other two strains. The rhizomorphs of A35 were thicker and more inclined to produce mycelium, while A19 displayed slower growth and less branching. These observations suggest that well-branched and thick rhizomorphs may be advantageous for cultivation and production. However, the limited number of *Armillaria* species studied here raises the question of whether other symbiotic *Armillaria* species, either from the same or different taxa, exhibit similar culture characteristics and consistent effects on the *P. umbellatus* yield. Further studies are needed to explore this possibility.

The ergosterol contents of A35-P, A541-P, and A19-P all met the standards specified in the ChP (2020), with no significant differences observed among the three groups. Ergosterol, a crucial component of fungal cell membranes, serves as a marker of fungal biomass [38] and indirectly influences fungal cell wall synthesis [39]. This suggests that differences in yield among A35-P, A541-P, and A19-P may also be related to variations in sclerotial density. In this study, the contents of polyporusterone A and polyporusterone B were significantly higher in A19-P than in A35-P and A541-P. Additionally, the accumulation of ergosterol, polyporusterone A, and polyporusterone B in A35-P, A541-P, and A19-P showed inconsistencies. These variations may be attributed to the conversion mechanism of ergosterol to polyporusterone A in *P. umbellatus*. Ergosterol can undergo hydroxylation to form a sixth hydroxylated product, which is subsequently oxidized to an α,β-unsaturated bond and transformed into a 2,14-dihydroxylated product via oxidative enzymes. This intermediate product, catalyzed by oxidative enzymes, forms a new unstable transition product with three oxygen bridges. This product is then converted into polyporusterone A with the participation of H^+^ ions [40]. The polysaccharides of *P. umbellatus* exhibit various biological functions, including anticancer activities, immune enhancement, and liver protection [14,41,42]. In this study, significant differences in polysaccharide contents were observed among A35-P, A541-P, and A19-P, with A541-P exhibiting the highest content. Although no official content threshold for polysaccharides in *P. umbellatus* exists, Lu proposed that the total polysaccharide content should not be less than 5% after analyzing 26 *P. umbellatus* samples [43]. All three groups in this study met the recommended value. The differing levels of polysaccharide accumulation among A35-P, A541-P, and A19-P may be related to variations in the activity of metabolic pathways in *P. umbellatus*, including polysaccharide synthesis, energy storage, and the expression levels of genes associated with polysaccharide metabolism [44]. Future studies should focus on further purification and characterization of the monosaccharide composition and sugar chain structure in these polysaccharides, as the monosaccharide composition plays a critical role in determining polysaccharide biological activity [45].

Our research demonstrated that the three symbiotic *Armillaria* strains had distinct effects on the yield and active compound contents of *P. umbellatus*. Among them, A541-P showed significant advantages in yield, ergosterol content, and polysaccharides, which are key indicators for assessing the yield and quality of *P. umbellatus* compared to A19-P and A35-P. Phylogenetic analysis revealed that A541 clustered with *A. gallica*, suggesting that *A. gallica* may hold significant potential for co-cultivation with *P. umbellatus*. Another study showed that 22 *Armillaria* strains were isolated from the sclerotium of *P. umbellatus*, of which 10 were *A. gallica*, also indicating that *A. gallica* might be a dominant species involved in the symbiosis of *P. umbellatus* [33]. Future studies could explore additional *Armillaria* species to validate these findings further or investigate whether *Armillaria* strains of the same species but from different sources affect the yield and quality of *P. umbellatus*. Similarly, differences in the species, origins, and phylogeny of *P. umbellatus* may also influence its yield and quality. However, an evolutionary classification of *P. umbellatus* variants or subspecies is currently lacking. Based on morphological differences, *zhushiling* and *jishiling* are generally recognized as the primary types of *P. umbellatus* [46]. This study focused on *zhushiling*, the most widely cultivated and medicinally used form of *P. umbellatus*. Further research is needed to examine how other *P. umbellatus* phylogenies affect its interaction with *Armillaria*. These findings have practical implications for selecting suitable *Armillaria* strains and achieving high-quality and abundant *P. umbellatus* in production. It is also noteworthy that traditional Chinese medicine (TCM) consists of numerous chemical components, with clinical efficacy resulting from their synergistic effects. A comprehensive evaluation of TCM using multiple indicators is therefore essential. In this study, we assessed the overall quality differences of *P. umbellatus* co-cultivated with different *Armillaria* species through PCA. The results highlighted that in addition to ergosterol, polyporusterone A, polyporusterone B, and polysaccharides are critical for the quality control of *P. umbellatus*.

## 5. Conclusions

This study is the first to demonstrate significant differences in the effects of three distinct symbiotic *Armillaria* strains on the yield, steroid content, and polysaccharide content of co-cultivated *P. umbellatus* sclerotia. These variations are likely attributable to the genetic and morphological differences among the *Armillaria* strains. Among the strains tested, A541, which is classified as *A. gallica*, showed the greatest potential for improving both the yield and quality of co-cultivated *P. umbellatus* sclerotia compared to A19 and A35. These findings provide a valuable reference for selecting symbiotic *Armillaria* strains in the artificial cultivation of high-quality and high-yield *P. umbellatus*.

## Figures and Tables

**Table 1:** Linearity, limit of detection, and limit of quantitation of the three types of reference substance.

Substance	Standard Curve	R^2^	Linear Range mg·mL^−1^	LOD ^1^ mg·mL^−1^	LOQ ^2^ mg·mL^−1^
ergosterol	y = 24322x − 70.7970	0.9990	0.0294–0.2058	5.948 × 10^−4^	1.487 × 10^−3^
polyporusterone A	y = 28316x + 15.0590	0.9999	0.0031–0.0408	6.272 × 10^−5^	1.568 × 10^−4^
polyporusterone B	y = 40549x − 12.3680	0.9992	0.0031–0.0408	8.981 × 10^−5^	2.245 × 10^−4^

^1^ LOD—limit of detection; ^2^ LOQ—limit of quantitation.

## Data Availability

All relevant data are within the manuscript and its additional files. The sequencing original data presented in the study are openly available in SUB2866618.
